# Turning a Pest into a Natural Enemy: Removing Earwigs from Stone Fruit and Releasing Them in Pome Fruit Enhances Pest Control

**DOI:** 10.3390/insects14120906

**Published:** 2023-11-24

**Authors:** Aldo Hanel, Robert J. Orpet, Richard Hilton, Louis Nottingham, Tobin D. Northfield, Rebecca Schmidt-Jeffris

**Affiliations:** 1 Department of Entomology, Washington State University, Pullman, WA 99164, USA; robert.orpet@wsu.edu (R.J.O.); louis.nottingham@wsu.edu (L.N.); tnorthfield@wsu.edu (T.D.N.); 2 Tree Fruit Research and Extension Center, Washington State University, Wenatchee, WA 98801, USA; 3 Southern Oregon Research and Extension Center, Oregon State University, Central Point, OR 97502, USA; richard.hilton@oregonstate.edu; 4 Northwestern Washington Research and Extension Center, Washington State University, Mount Vernon, WA 98273, USA; 5 Temperate Tree Fruit and Vegetable Research Unit, United States Department of Agriculture-Agricultural Research Service, Wapato, WA 98951, USA; rebecca.schmidt@usda.gov

**Keywords:** aphids, biological control, earwig, IPM, pear psylla, trapping, tree fruit

## Abstract

**Simple Summary:**

Biological control, which is the use of natural enemies to regulate crop pests, is considered a key aspect of integrated pest management. In pome fruit (apple and pear) crops of the northwestern United States, the European earwig *Forficula auricularia* (L.) sensu lato may provide a unique opportunity for augmentative biological control. While this generalist omnivore is a direct pest in stone fruit crops like cherries and peaches, it is a beneficial predator in apple and pear crops. In these crops, it feeds on two key pests, the woolly apple aphid and pear psylla, respectively, and rarely damages fruit. Here, we tested a two-way strategy to reduce fruit damage by removing earwigs from crops where they are pests and releasing earwigs into orchards where they can help control pest populations. We found that mass-trapping earwigs in stone fruit orchards did not significantly reduce earwig numbers or fruit injury; however, this was a relatively easy and practical method for collecting thousands of individuals for augmentative release in pome fruit orchards. Two mass releases (once annually) of earwigs helped control key pests in pears and apples, such as pear psylla and woolly apple aphids, by the second year. We did not find evidence of a reduction in other pests, such as mites and other aphid species. Finally, we found that earwig releases can be useful in establishing their populations in orchards where they are absent or rarely found, potentially providing increased pest control over multiple seasons.

**Abstract:**

The European earwig *Forficula auricularia* (L.) (Dermaptera: Forficulidae) is an omnivorous insect that is considered a minor pest of stone fruit and a key predator of pests in pome fruit orchards. In many pome fruit orchards, earwigs are absent or in low abundance due to broad-spectrum spray programs and the slow recolonization rate of earwigs. Orchards in transition to organic or “selective” conventional programs often struggle to achieve effective levels of biological control, and thus, may benefit from inoculating earwigs to expedite their re-establishment. In a two-year study, we evaluated the potential for mass trapping earwigs from stone fruit using rolled cardboard traps to reduce fruit damage and provide earwigs for augmentation in pome fruit. We also tested whether a single mass release or five releases (on alternating weeks) of the same total number of earwigs in apples and pears reduced pests relative to plots where no releases occurred. Mass trapping did not decrease earwig abundance or substantially reduce fruit damage in stone fruit orchards. However, trapping was an efficient method for providing earwigs for augmentation. Earwig abundances were only increased in orchards where earwigs were previously low or absent; however, multiple orchards with varying prior levels of earwigs exhibited reductions in key pests (woolly apple aphid and pear psylla). For some other pests evaluated, plots with mass releases of earwigs had a slight trend in overall lower pest density when compared with control plots. A strategy for moving earwigs out of stone fruit orchards and into pome fruit orchards could be an effective method for augmenting orchard predator populations.

## 1. Introduction

Biological control, the use of natural enemies to regulate pest populations, is a key component of integrated pest management (IPM) [1]. Worldwide, tree fruit orchards heavily rely on biological control and implement conservation practices such as limiting broad-spectrum pesticide use or adding beneficial plants. This often results in increased natural enemy abundance and reduced pest pressure [2,3,4]. For apples in the Pacific Northwest (PNW) region of the United States, conservation of natural enemies with selective pesticide use saves an estimated USD 519/ha [5]. Conservation practices are not as widely adopted in PNW pears [5] but eliminating the use of pesticides that are high risk to natural enemies has been demonstrated to increase natural enemy abundance [3,6]. Following these newer IPM recommendations for pears also results in similar or lower pear psylla (*Cacopsylla pyricola* (Foerster)) levels compared with conventional practices while simultaneously decreasing insecticide applications [6].

The European earwig (*Forficula auricularia* L.), sensu lato [7], is a widely distributed omnivorous insect known to feed both on plant and animal material [8,9]. Its role as a key predator of important pests in the PNW has not always been widely appreciated, possibly because its nocturnal foraging behavior causes it to be missed by orchardists scouting during the daytime [10]. Earwigs are effective predators of the woolly apple aphid, *Eriosoma lanigerum* (Hausmann) [10,11,12,13,14,15], and the rosy apple aphid, *Dysaphis plantaginea* (Passerini) [16]. When earwigs are added to apple orchards, aphid populations decrease [10,12]. Earwigs also show potential for controlling pear psylla (*Cacopsylla pyri* L.) in Europe [17,18] and *C. pyricola* in the United States [19]. European earwigs can also consume other apple and pear pests, including codling moth, *Cydia pomonella* L., the most important pest of apples worldwide [20]. Orchards where earwigs are absent tend to have higher aphid, pear psylla, and codling moth populations [21].

In temperate climates, earwigs only produce one generation per year, making their populations highly prone to disruption by pesticides or other factors of mortality, such as soil tillage [9,14,22,23]. Because earwigs overwinter below the soil surface and have a nesting and ground-free-foraging stage, they are particularly vulnerable to soil management practices [9]. However, in temperate tree fruit crops, tillage typically occurs in the summer, where earwigs are less vulnerable to soil management because late-instar nymphs and adults have transitioned from the ground-foraging phase into the tree canopy [9]. Earwigs are also poor dispersers and rarely fly [24]. Therefore, orchards that are transitioning away from broad-spectrum spray programs, which eliminated endemic earwig populations, may benefit from inoculating earwigs to expedite their re-establishment. Effective earwig traps can be made at a very low cost using rolled corrugated cardboard, which is attached to the tree [10,25,26]. The traps are attractive due to the earwigs’ preference for tight spaces and are likely made more attractive because earwigs use aggregation pheromones [27,28]. Although rearing earwigs in the laboratory is possible [12,16,29], this does not provide an efficient source for augmentation due to their univoltinism and specialized nesting requirements. This long generation time also means that earwigs will likely not be profitable for rearing in commercial insectaries, especially because there is likely to be minimal demand outside of orchard crops. Despite this lack of supply, there are consistent requests from PNW orchard managers for sources of earwigs for augmentation programs (Schmidt-Jeffris, pers. obs.). Therefore, growers have a need for a source of earwigs for augmentation that is currently not being met by commercial supply.

Although they are omnivorous, earwigs do not cause economically relevant damage to the leaves of mature trees or to pome fruits [8,9]. Although reports of earwigs damaging apples exist, this is likely caused by opportunistically using previous existing damage (e.g., stem bowl split, birds, hail), which would have resulted in a culled fruit, regardless of the expansion of damage by secondary earwig feeding [9,10]. However, earwigs are considered a minor pest of stone fruit (e.g., cherries, apricots, plums) because they can perforate the fruits’ soft flesh [8,9,30], causing, for example, damage of up to 40% in apricots [31]. Because many orchard operations in the PNW grow both pome fruit and stone fruit, this presents a unique opportunity. Pome fruit orchards where earwigs are absent could potentially obtain earwigs from stone fruit orchards and use them for inoculation/augmentative biological control. In other biological control efforts involving moving predators into pest outbreak areas (usually involving lures), there is a concern about the ‘robbing Peter to pay Paul’ effect (i.e., moving the predator causes a new outbreak in the area where they are moved from) [32,33]. Sourcing earwigs from stone fruit would avoid this issue. Furthermore, if repeated, mass trapping is used, there may be an added benefit of reducing earwig abundance in stone fruit orchards, resulting in decreased damage.

The objective of this study was to develop mass-trapping and augmentation protocols for earwigs in PNW stone fruit and pome fruit, respectively. In Washington State and Oregon, we conducted a two-year study to test (1) whether mass trapping was a reliable strategy to obtain large amounts of earwigs and (2) whether mass trapping reduced earwig populations and fruit damage in stone fruit. We also examined whether earwig releases in pome fruit (3) helped establish and increase earwig abundance and (4) whether this had an effect on pest control.

## 2. Materials and Methods

### 2.1. Earwig Mass-Trapping: Field Sites and Experimental Design

To determine if mass-removing earwigs from orchards reduced their populations over time, we monitored and captured earwigs using corrugated cardboard traps in the summers of 2021 and 2022. Each trap consisted of a 10 cm (width) × 30 cm (length), single-sided corrugated cardboard strip (ULINE, Pleasant Prairie, WI) rolled into a cylinder and taped together. The experiment was conducted at one apricot and one cherry orchard in Washington State (WA) (Table 1). Pesticide records for all orchards used each year are provided in the Supplementary Materials (Table S1). The experiment consisted of six (apricots) or five (cherries) replicates per treatment. Replicates were 16-tree plots (4 rows × 4 trees down a row), totaling 0.0072 ha and 0.0128 ha in apricots and cherries, respectively. Each individual tree had one cardboard trap attached ~80 cm from the ground, either on the trunk, the main limbs, or the first branches coming off the main limbs, depending on tree architecture and size, using flagging tape. Replicates were spaced at least 30 m apart to minimize within-season dispersal of earwigs between plots [34]. We had two treatments: a control, where earwigs were counted weekly and returned to their respective trees, and a removal treatment, where earwigs were counted weekly and removed from the orchard. Earwigs were counted by removing the cardboard trap from the tree and emptying the trap into a bucket. Cardboard traps were re-used each time, and only replaced if lost or destroyed (e.g., hit by sprinklers or machinery). Weekly sampling was conducted from 25 May to 9 August 2021 and 11 May to 9 September 2022. Sampling was always performed during the morning using the same plots in both years.

### 2.2. Earwig Mass-Trapping: Damage to Stone Fruit

To determine if mass removing earwigs from stone fruit orchards reduced fruit damage, we conducted a fruit sample near harvest time in these plots. Apricots were sampled on 16 June 2021 and 12 June 2022. Cherries were sampled on 22 June 2021 and 7 June 2022. We sampled 32 apricot fruits (~2 per tree) and 64 cherry fruits (~4 per tree) and visually assessed possible earwig damage. Fruits were examined for wounds that appeared to be caused by chewing. Due to the difficulty of differentiating earwig damage from that of other insects and birds, these numbers are likely overestimates but serve to determine if there are differences in damage between treatments.

### 2.3. Earwig Maintenance after Capture

Earwigs captured in stone fruit were brought to the laboratory, kept in large plastic bins (~62 L) with wet cotton balls to provide moisture, and fed with dog kibble (Wellness Core, Wellness Pet; Tewksbury, MA, USA) and, every other week, with apricot and cherry fruits and leaves from the orchards used in the mass-trapping experiment. The containers were kept in a growth room set to a 16:8 L/D light cycle and 22 °C until the earwigs were used for the releases. The earwigs were used in the earwig-augmentation experiment described below within the same year that they were captured.

### 2.4. Earwig Augmentation: Field Sites and Experimental Design

To determine if introducing earwigs in pome fruit orchards increases their abundance over time, we released the earwigs collected from the mass-removal experiment into new orchards and monitored the earwigs using corrugated cardboard traps with sampling taking place early in the morning. Replicated experiments were conducted at three sites: two pear orchards (one each in WA and Oregon (OR)) and one WA apple orchard (Table 1). The soil and ground cover management of the three orchards was similar and typical for northwestern U.S. pears: all had an orchard grass ground cover in the row middles, which was periodically mowed. A relatively bare strip under the trees was maintained with a mechanical cultivation weeder (Wonder Weeder, Burbank, WA, USA) and flame-treatment in the Washington orchards (a common practice in organic orchards), whereas the Oregon pear orchard maintained the strip using pre-emergent and burndown herbicides (Table S1).

We had three treatments: (1) continuous release, (2) mass release, and (3) a no-release control. Each orchard site consisted of five replicates of each treatment, each consisting of nine trees (three trees down a row, three rows), at least 10 m away from the orchard edge, where earwigs were released in the central tree. Replicates were blocked by location within the orchard and spaced at least 30 m apart. In the continuous release, we released 100 earwigs every other week for ten weeks during the season (a total of 500 earwigs released per plot). In the mass release, 500 earwigs per plot were released, once early in the season on the same date as the first “continuous release” in each orchard. For the first date of release for each orchard, see Table 2. Releases occurred in both years of the study (2021–2022), using the same plots each year. 

The day before release, earwigs were removed from the holding containers and transferred to plastic bags (~946 mL) containing one cardboard trap to provide shelter, dog kibble, and one wet cotton ball to provide moisture, with either 100 or 500 earwigs per bag, depending on the treatment. The trap and earwigs within each bag were then placed on the appropriate release tree the following morning by tying the trap to the tree (as in Section 2.1) and then shaking any earwigs not in the trap out of the bag directly onto the tree. 

### 2.5. Earwig Augmentation: Establishment after Release

Each tree within a plot was monitored with one cardboard trap placed on the tree ~80 cm from the ground. Traps were checked once per month by emptying earwigs into a bucket, counting them, and then returning the trap and earwigs to the same tree. Sampling occurred June–August in 2021 and June–September in 2022 (Table S2).

### 2.6. Earwig Augmentation: Pest Monitoring

We monitored the abundance of the target pest species in the apple and pear orchards where earwigs were released. In the apple orchard, aphids were monitored by examining four random 30 cm shoots per tree and counting the number of infested leaves (green apple aphid, rosy apple aphid) or the number of distinct colonies on a shoot (woolly apple aphid) [10,35]. This was performed every other week from 21 June to 16 August 2021 and 21 June to 30 September 2022. For pears, we collected samples of 27 leaves per plot (3 randomly selected leaves per tree) every other week from 17 June to 10 August 2021 and 21 June to 30 September 2022. Leaf samples were brought to the laboratory for processing. Leaves from a plot were brushed onto soap-coated glass plates using a mite brushing machine (Leedom Enterprises, Mi-Wuk Village, CA, USA). We counted the number of *C. pyricola* (eggs, small nymphs: 1st-3rd instars, and large nymphs: 4th–5th instars), eggs and motiles of spider mites (two-spotted spider mite, *Tetranychus urticae* Koch; brown mite, *Bryobia rubriocolus* Koch; European red mite; *Panonychus ulmi* (Koch)), and pear rust mite (*Epitrimerus pyri* Nalepa) motiles on each glass plate using a stereomicroscope. 

### 2.7. Data Analysis

All analyses were conducted using R software, version 4.1.3 [36]. Before fitting a model, data were checked for zero inflation and overdispersion using the package DHARMa [37]. Pre- and post-release (all dates combined) count data were analyzed separately.

#### 2.7.1. Earwig Mass-Trapping

Data were analyzed separately by year and by orchard with a generalized linear mixed model (GLMM) using the package glmmTMB [38], with earwig counts as the dependent variable, treatment as a fixed factor, and statistical block and sampling date as random factors (to account for repeated measures), with a negative binomial error distribution (nbinom1) and log link function. Earwig stone fruit damage data were analyzed separately by year and by orchard using a GLMM with the package lme4 [39], with damage (number of fruit damaged/total) as the dependent variable, treatment as a fixed factor, and replicate as a random factor, with a binomial error distribution and logit link function.

#### 2.7.2. Earwig Augmentation

Earwig and pest data were analyzed separately per year and per orchard using a generalized linear mixed model (GLMM) using the package glmmTMB [38], with either earwig or pest species counts as dependent variables, treatment as a fixed factor, and statistical block and time as random factors, specifying a Poisson (earwigs in pears (OR), woolly apple aphids, green apple aphids (2022), rosy apple aphids (2021), pear psylla, two-spotted spider mites, pear rust mites (2021, WA), brown mites (2021, WA), and European red mites (WA)) or negative binomial (nbinom1 prompt in package glmmTMB [38]) (earwigs in apples (WA) and pears (WA), green apple aphids (2021)) distribution based on the results of zero-inflation and overdispersion tests. 

## 3. Results

### 3.1. Earwig Mass-Trapping

**Figure 1:**
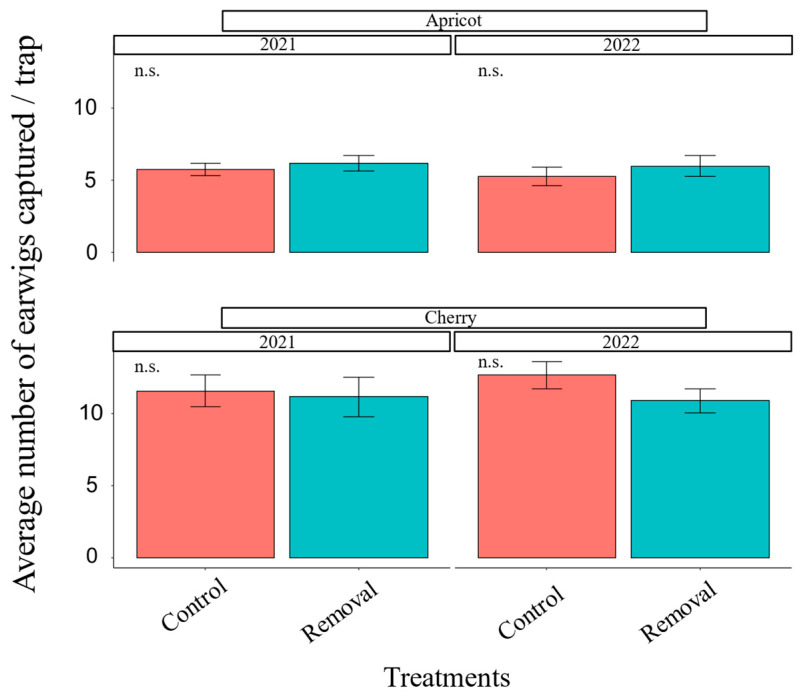
Annual mean number of earwigs (±SE) captured per trap separated by crop and year. n.s. = non-significant at *p* < 0.05 (GLMM).

**Figure 2:**
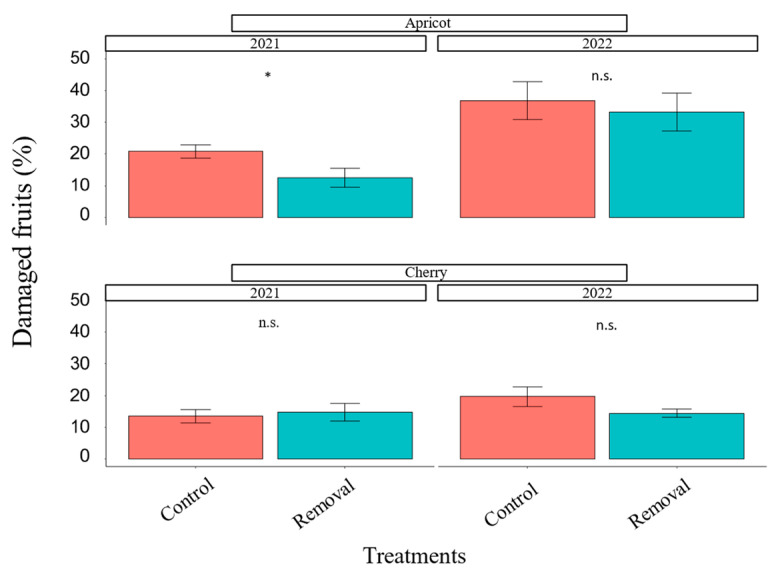
Percentage of fruits with possible earwig damage per crop per year. An asterisk (*) indicates that treatments differ within the same year and crop at *p* < 0.05 (GLMM). n.s. = non-significant at *p* < 0.05.

There was no significant difference in mean seasonal earwig abundance between the mass-trapping treatment and the control in either crop in either 2021 or 2022 (Figure 1). There was a significant reduction in possible earwig damage in apricots in 2021 (*χ^2^* = 4.71, df = 1, *p* = 0.03; Figure 2) but not in 2022 (*χ^2^* = 0.26, df = 1, *p* = 0.6; Figure 2). However, in the apricot orchard in 2021, we were only able to sample fruits after harvest started. This may have affected results by causing us to sample a higher portion of damaged fruits intentionally left on the tree by pickers. In cherries, we did not observe any significant difference in fruit damage between the removal and control plots in 2021 (*χ^2^* = 0.18, df = 1, *p* = 0.73; Figure 2) but found weak evidence of damage reduction in 2022 (*χ^2^* = 2.96, df = 1, *p* = 0.08; Figure 2).

### 3.2. Earwig Augmentation

**Figure 3:**
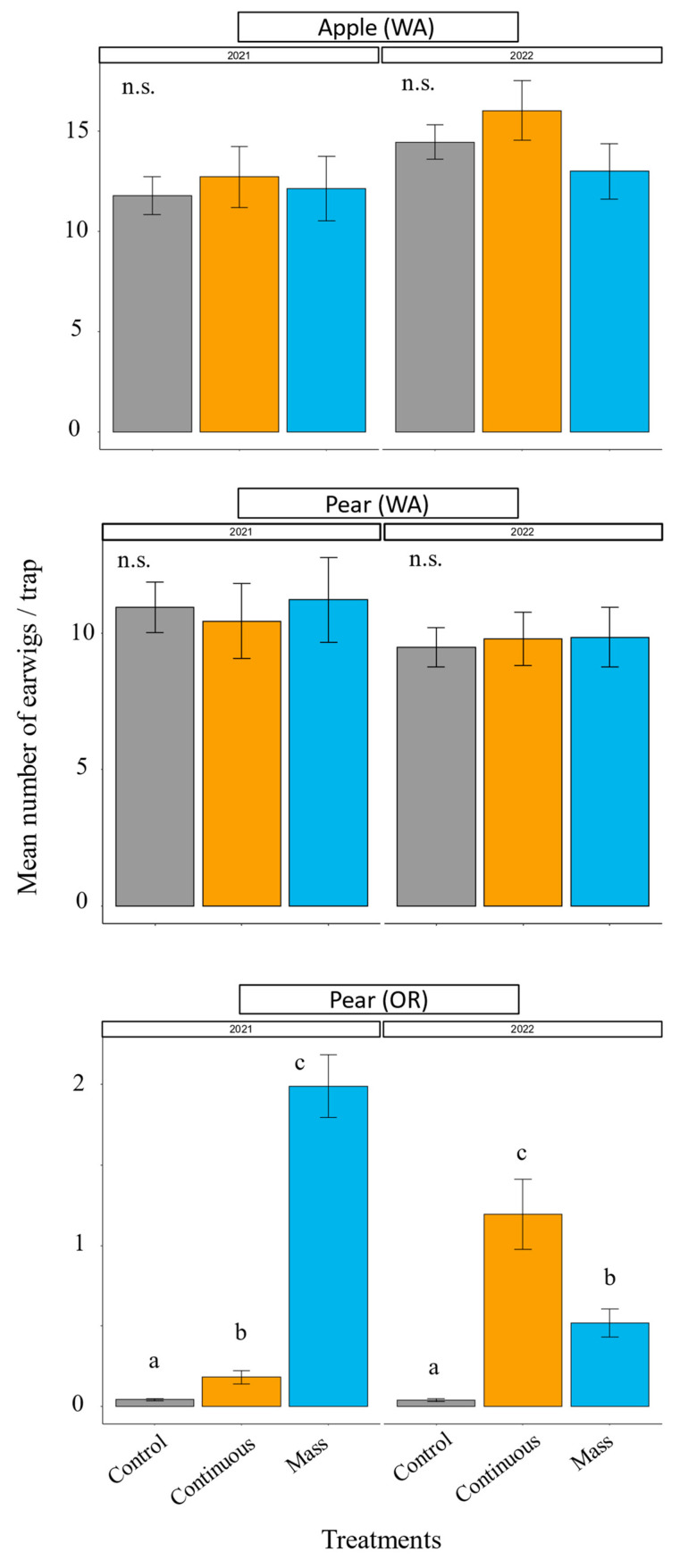
Annual post-release mean number of earwigs (±SE) captured per trap separated by crop, location, and year. Different letters above the bars indicate that treatments differ significantly within the same year, crop, and location at *p* < 0.05 (contrast after GLMM). n.s. = non-significant at *p* < 0.05.

Earwig abundance prior to the releases varied between locations. Both apple and pear orchards in WA had high earwig abundance prior to the releases, whereas the pear orchard in OR had no earwigs before the releases in 2021 (Table S3). In the WA apple orchard, earwig abundance in the treated plots, either with one mass release or continuous releases, did not differ from the control in 2021 (*χ^2^* = 0.04, df = 2, *p* = 0.9779; Figure 3) or 2022 (*χ^2^* = 1.46, df = 2, *p* = 0.4816; Figure 3). We observed the same trend in the WA pear orchard: earwig abundance did not differ between the treatments in 2021 (*χ^2^* = 0.27, df = 2, *p* = 0.8732, Figure 3) or 2022 (*χ^2^* = 0.53, df = 2, *p* = 0.7643, Figure 3). In the OR pear orchard in 2021, the release treatments had significantly higher earwig abundance compared with the control and also differed from each other (*χ^2^* = 317.64, df = 2, *p* < 0.001, Figure 3). Compared with the control, the mass release had 49× more earwigs and the continuous release had 4× more earwigs (Figure 3). In 2022, there was a low number of earwigs in the OR pear control plots (Table S3). There was a significant effect of the earwig release treatment in the OR pear orchard in 2022 (*χ^2^* = 77.73 df = 2, *p* < 0.001, Figure 3). All treatments were significantly different from each other, with continuous release having higher overall earwig abundance, followed by the mass release and then the control (Figure 3).

### 3.3. Earwig Augmentation: Pest Control

**Figure 4:**
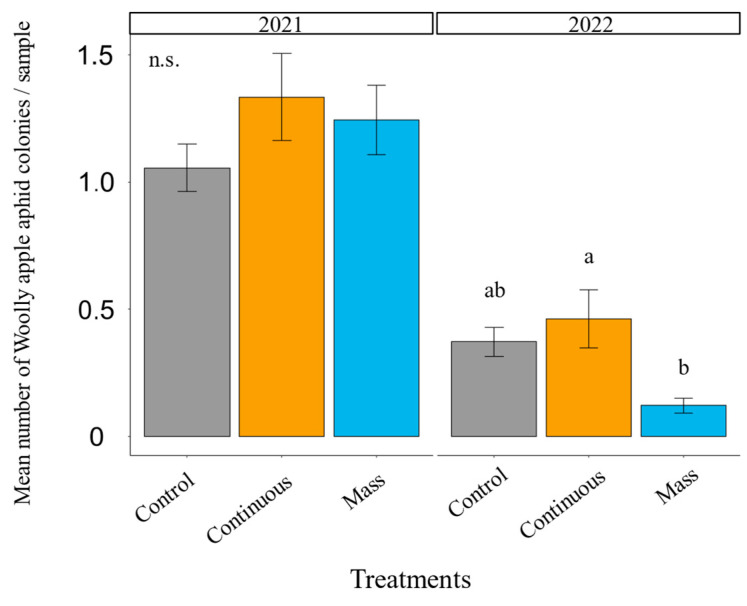
Annual post-earwig release mean number of woolly apple aphid colonies (±SE) per sample separated by year in the WA apple orchard. Different letters above the bars mean that treatments differ significantly within the same year at *p* < 0.05 (Contrast after GLMM). n.s. = non-significant at *p* < 0.05.

The woolly apple aphid was more abundant than the other two aphid species (green apple and rosy apple aphids) in both years in the WA apple orchard (Table S4). The woolly apple aphid and green apple aphid populations were higher in 2021 than in 2022, while the rosy apple aphid abundance was similar each year. We observed significant differences in the number of woolly apple aphid colonies between treatments in 2022 (*χ^2^* = 6.81, df = 2, *p* = 0.0331, Figure 4 and Figure S1) but not in 2021 (*χ^2^* = 2.21, df = 2, *p* = 0.331, Figure 4 and Figure S1). In 2022, the mass-release treatment had the lowest number of woolly apple aphid colonies, differing significantly from the continuous release (*p* = 0.0488, Figure 4), and was marginally different from the control (*p* = 0.0507, Figure 4). Numerically, the mass-release treatment had 61% fewer woolly apple aphids than the control. Green and rosy apple aphid counts did not differ between treatments in either year of this study (Table S4).

**Figure 5:**
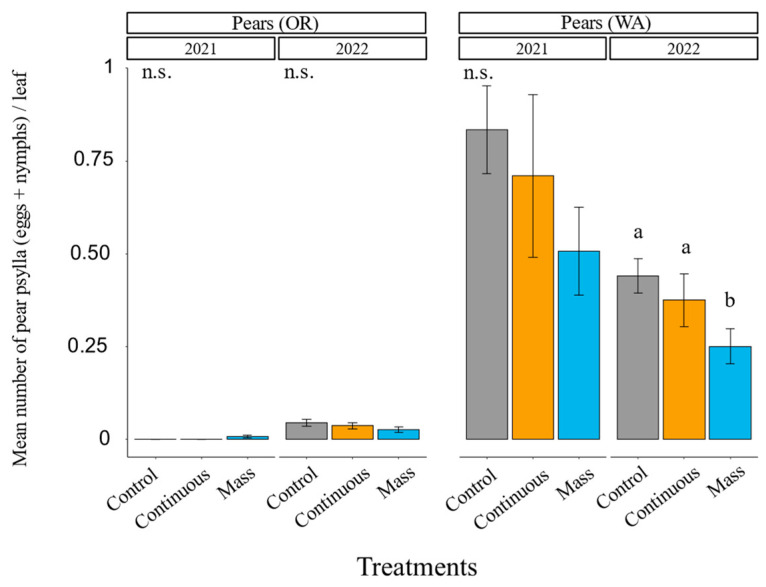
Annual post-earwig release mean number of pear psylla (eggs + nymphs) (±SE) per leaf separated by location and year. Different letters above the bars indicate that treatments differ significantly within the same location and year at *p* < 0.05 (contrast after GLMM). n.s. = non-significant at *p* < 0.05.

In the WA pear orchard, pear psylla populations were higher in 2021 than in 2022. We observed a significant reduction in pear psylla (eggs and nymphs combined) in 2022 (*χ^2^* = 8.59, df = 2, *p* = 0.0136, Figure 5 and Figure S2) but not in 2021 (*χ^2^* = 3.25, df = 2, *p* = 0.1966, Figure 5 and Figure S2). Numerically, the control had the highest pear psylla counts each year, followed by the continuous release and then the mass release (Figure S2). In 2022, there were significantly fewer pear psylla in the mass-release treatment compared with the control and the continuous release, which did not differ from each other (Figure S2). Abundance of all three spider mite species was higher in 2021 than in 2022. European red mites were the most abundant, followed by brown mites and then two-spotted spider mites. We did not find a significant effect of the treatment on two-spotted spider mite, European red mite, pear rust mite, or brown mite abundances in either year (Table S4). There was a numerical trend toward fewer pest mites in the mass release compared with the control in this orchard (Table S4).

In the OR pear orchard, there was not a significant reduction in pear psylla abundance in either 2021 (*χ^2^* = 0, df = 2, *p* = 1, Figure 5) or 2022 (*χ^2^* = 3.48, df = 2, *p* = 0.1747, Figure 5). There was no significant difference between the treatments in the two-spotted spider mite and pear rust mite abundance in either 2021 or 2022 (Table S4). Pest populations were much lower in this pear orchard than in the WA pear orchard.

## 4. Discussion

Our results provide evidence of the potential of European earwigs as an important tool for integrated pest management in tree fruit orchards. This is a novel trap-and-move protocol for an arthropod from crops where it is a pest to crops where it is beneficial. Released earwigs increased suppression of multiple pests of pome fruit orchards, supporting other studies that have confirmed this for single pests [10,12,16,18]. Mass-trapping earwigs in stone fruit orchards was not successful as a pest control tactic, but it did provide large numbers of earwigs for augmentation purposes in pome fruit. The trend in slight damage reduction may indicate that a longer mass trapping program, or one that uses more of the orchard, might be successful. Releasing earwigs in a single, early-season mass release reduced the abundance of woolly apple aphids and pear psylla; single, annual releases would also likely be more convenient for growers. Earwig releases increased earwig abundance within one season, but only in the OR pear orchard where they were absent prior to releases. The use of corrugated cardboard is a well-established monitoring technique for earwigs [9], and its use as a pest control method is recommended by extension materials for home use, with or without the aid of baits [40,41]. However, to our knowledge, no field trials have investigated mass trapping as a pest control tactic for earwigs. Instead, previous research has demonstrated that excluding earwigs from the canopy using adhesive barriers can reduce earwig abundance in the canopy and decrease damage to apricots [42] and cherries [30]. In our study, the mass removal of earwigs using corrugated cardboard traps was not consistently successful in reducing fruit damage for either crop. Preventing earwigs from reaching fruits may be more important for damage reduction than reducing populations, although achieving both can improve results [30,42]. However, future studies are needed to evaluate mass trapping for a longer term and/or across a larger area to truly determine if this tactic can reduce earwig damage. Although earwigs are poor dispersers, they can likely move into the edges of removal plots, resulting in no observed differences between treatments in our study. Repeating removal across multiple years may also slowly reduce the entire population, which we may have observed the beginnings of in the cherry orchard in 2022. 

In terms of increasing earwig abundance, earwig releases might not be as effective in orchards where earwig populations are high compared with orchards where they are virtually absent. Orpet et al. [9] proposed the use of European earwig augmentation only in pome fruit orchards where earwig populations were low or undetectable because the pest control service provided by additional earwigs has minimal returns where they are already abundant (see also [16,43]). In our orchard with low earwig abundance (OR), the releases resulted in earwig establishment; they began to be observed in the control plots only after releases began and were found in 2022 prior to the start of releases that year. Given that earwigs were not present in these plots prior to the releases in 2021, this likely indicates that earwigs from released plots had begun to move into control plots. Overall numbers in the OR orchard were still very low even after two years of releases (seasonal average of one per trap at most), suggesting that yearly releases might still be needed to achieve thresholds for pest control. It has been suggested that greater than two earwigs per trap is correlated with the suppression of second- and third-generation pear psylla [19]. Another argument in favor of yearly releases in orchards with low earwig abundance is that due to their poor dispersal behavior—moving only up to 30 m per month [34]—earwigs are thought to slowly recolonize areas where they have been absent [9]. Other factors have also been shown to improve earwig establishment and abundance, such as avoiding certain pesticides [9,43]. For orchards with either high or low previous earwig abundance, one early-season mass release seems to be preferable to continuous releases: we observed that a single release early in the season in the OR pear orchard had greater earwig establishment and fewer pear psylla compared with the continuous-release treatment. 

In pears, most previous research with earwig augmentation has been conducted in Europe, targeting the pear psylla species *C. pyri* [17]. This research demonstrated that increasing earwig abundance could reduce *C. pyri* abundance [18,44]. Regarding *C. pyricola*, we showed that earwig releases can significantly reduce their abundance, especially when using one mass release of earwigs. Earwigs are considered a resident predator due to their biology—univoltine, poor dispersal, broad diet [9]—they tend to stay in the area even when prey numbers are low. Therefore, as is typical for density-independent control [45], it is expected that they are more likely to prevent increases in pest populations, rather than decrease the abundance of already problematic pest populations [46]. This partially explains why earwigs, as an important part of the natural enemy complex of pear psylla in the Pacific Northwest, assist mostly in controlling a pest during their second and third generations [19]. 

Our results only showed pear psylla reductions after releases in our WA pear orchard (not the OR orchard) and only in 2022 but not in 2021. One possible explanation for this could be that the OR pear orchard had consistently low pear psylla abundance—possibly because this orchard is conventionally managed—substantially reducing our ability to detect differences in pest levels. In 2022 in OR, although not significant, we did observe a similar trend in our results when compared to what we observed in WA in 2022; there were fewer pear psylla in the mass-release treatment compared with the continuous release and the control. It is possible that in an orchard with no earwigs prior to releases (like our OR orchard) but with higher pest densities (like our WA orchard), differences in pear psylla abundance between treatments would be more dramatic. The efficacy of earwigs in controlling pear psylla is also subject to outside factors, mainly pesticide use patterns [18], as well as ground cover management practices, the presence of natural enemies and pathogens, and overwintering mortality [9].

Our results agree with previous studies on apples, demonstrating that earwigs can reduce woolly apple aphid populations. The efficacy of earwigs in controlling woolly apple aphids seems to be highly dependent on factors such as plant architecture, both current pests and earwigs, and the abundance of other natural enemies [10,15,47,48,49]. Apple tree shoots with intermediate and high abundance of earwigs reduced woolly apple aphid infestation from 30–35% to 10%, when compared with shoots where earwigs were excluded [47]. Earwig augmentation, especially at low aphid densities, helped suppress woolly apple aphid population growth throughout the season [10]. The number of earwigs present and the current woolly apple aphid abundance, especially around June, also have a key role in the success of aphid control with earwigs for the remainder of the season [48,49]. For instance, simulations suggested a mean wooly apple aphid-to-earwig ratio of 50 for successful aphid control with earwigs [48], while another study suggested that orchards with >14 earwigs/trap early in the season have lower aphid abundance [49]. Another important aspect of successful earwig biocontrol of woolly apple aphids is the pesticide regime—with success found only in IPM-managed orchards, but not in conventionally-managed ones, where broad-spectrum insecticides likely cause earwig mortality [43]. Finally, our results showed that one mass release of earwigs earlier in the season seems to be preferable over multiple releases. This could be the result of the previously discussed resident predator characteristics, where earwigs can help control aphid population increases but hardly reduce high populations [46].

Although no significant reduction was observed, there were numerically fewer green apple aphid populations (but not rosy apple aphids) in plots with one mass release of earwigs compared with the control. In previous studies, earwig inoculation in three-year-old apple trees reduced green apple aphid populations ten-fold when releasing five to six earwigs per tree [12], although no evidence of suppression was found in a follow-up experiment using six-year-old apple trees with four earwigs released per tree [15]. However, for rosy apple aphids, previous studies have shown that earwigs are important natural enemies—especially if present earlier in the season [50]—and can prevent aphid population increases [16]. In our study, possible explanations for a lower effect of earwigs in suppressing green and rosy apple aphids include the low overall pest pressure in our WA apple orchard but also the effects of ant-tending. Green and rosy apple aphids are tended by ants, reducing the efficacy of natural enemies, while woolly apple aphids are not [51]. 

It is notable that we only observed treatment differences in pest density in orchards where earwig density did not differ between treatments (WA apple and WA pear), whereas there were no differences in pest density where earwig density did differ between treatments (OR pear). While low pest densities in the OR pear orchard may explain the latter result, the former result is less clear. It is possible that earwig populations increased after releases in the WA orchards, but our study was unable to measure what would be a relatively less dramatic change in abundance because earwig populations were already high in these orchards. This could be due to trap “saturation” or the earwigs redistributing themselves either within the tree (not in the traps) or within plots post-release. It is also possible that, especially in the case of the mass-release treatment, earwig releases may have quickly depleted pest populations at the single-tree scale we were measuring and then moved on to a nearby tree that we were not monitoring. More research will be needed to develop a better understanding of earwig dispersal post-release and how earwigs respond to changing prey densities to draw clear conclusions regarding what trap counts of released earwigs may mean for the success of a biological control effort.

## 5. Conclusions

While earwigs, as generalists and omnivores, are not a silver-bullet biological control solution for managing key pests, augmenting and conserving their populations in tree fruit orchards has the potential to be an important new tool for improving sustainable fruit production. It is still unknown what would be the ideal release rate and timing for earwigs, although we showed evidence that early-season mass releases may be preferable to multiple introductions. This is likely due to earwigs helping prevent pest population increases, rather than controlling already abundant pests. Meanwhile, thresholds for pest control by earwigs have been developed for woolly apple aphids and pear psylla, e.g., [10,19,43], which, alongside earwig monitoring, can provide growers with insights on the need for earwig augmentation. Timing for the proper trapping of earwigs in stone fruit is also a key part of earwig augmentation programs. Phenology models, e.g., [52], could help growers decide when to invest labor and resources in deploying cardboard traps to maximize earwig catches during early-season peak earwig populations. Future studies should also focus on the ecological effects of earwig augmentation on native arthropod communities and the importance of timing releases properly to avoid intraguild interactions and other negative indirect effects.

Our study demonstrates that biocontrol practitioners can make a natural enemy out of a pest using a trap-and-move approach between crops. Omnivorous and generalist predators have many advantages for biological control, such as feeding on alternative food when prey numbers are low, reducing their dispersal rate and facilitating establishment in the crop [53], or activating plant defenses through phytophagy and indirectly impacting pests [54]. Other omnivorous insects may also be good candidates for “trap-and-move” biological control, such as the hemipteran *Campylomma verbasci* (Meyer-Dür), which is considered a pest of apples but is an important predator of pear psylla in pears [55]. There are likely other crop systems where a pest can become a predator by changing its location, potentially yielding a dual pest control benefit.

## Figures and Tables

**Table 1:** Characteristics of orchards used in both experiments.

Experiment	Site Location	Crop	Cultivar	Management Type	Row Spacing (m)	Tree Spacing (m)
Mass trapping	Yakima Co., WA, USA	Apricot	Unknown	Conventional	3	1.5
Mass trapping	Benton Co., WA, USA	Cherry	Chelan	Organic	4	2
Augmentation	Yakima Co., WA, USA	Pear	Bartlett	Organic	6	6
Augmentation	Jackson Co., OR, USA	Pear	Comice	Conventional	4.5	2.3
Augmentation	Yakima Co., WA, USA	Apple	Pink Lady	Organic	3	0.5

**Table 2:** First date of earwig releases in apple and pear orchards in 2021 and 2022.

	First Date of Earwig Release	
Orchard	2021	2022
Apple—WA	21 June	28 June
Pear—WA	17 June	21 June
Pear—OR	7 June	8 June

## Data Availability

The data presented in this study are available in the attached file Database S1.

## Supplementary Materials

The following supporting information can be downloaded at: https://www.mdpi.com/article/10.3390/insects14120906/s1. Figure S1. Mean number of woolly apple aphid colonies (±SE) per sample over time separated per year. Arrows indicate the release dates of the single mass release (blue arrow) or each continuous release (orange arrows); Figure S2: Mean number of pear psylla (eggs + nymphs) (±SE) per leaf over time separated per year. Arrows indicate the release dates of the single mass release (blue arrow) or each continuous release (orange arrows).; Table S1: Pesticide records for all orchards used for mass trapping and augmentation experiments during the time the experiment took place; Table S2: Earwig sampling dates for all orchards used in the augmentation experiment; Table S3: Mean number of earwigs, pear psylla (eggs + nymphs), and woolly apple aphid colonies before earwig releases started for both years. All means are followed by (±SE); Table S4: Mean number of twospotted spider mites, pear rust mites, European red mites, and brown mites in pears, and mean number of green apple aphid and rosy apple aphid colonies in apples in 2021 and 2022. All means are followed by (±SE). Data S1: Database S1.
