# Supplementary material for: Turning a Pest into a Natural Enemy: Removing Earwigs from Stone Fruit and Releasing Them in Pome Fruit Enhances Pest Control
Source: Insects. 2023 Nov 24;14(12):906. doi: 10.3390/insects14120906 (PMC10743910; doi:10.3390/insects14120906)

Supplemental tables

Table S1. Pesticide records for all orchards used for mass trapping and augmentation experiments during the time the experiment took place.

Table S2. Earwig sampling dates for all orchards used in the augmentation experiment.

Table S3. Mean number of earwigs, pear psylla (eggs + nymphs), and woolly apple aphid colonies before earwig releases started for both years. All means are followed by (±SE).

Table S4. Mean number of twospotted spider mites, pear rust mites, European red mites, and brown mites in pears, and mean number of green apple aphid and rosy apple aphid colonies in apples in 2021 and 2022. All means are followed by (±SE).

Supplemental figures

Figure S1. Mean number of woolly apple aphid colonies (±SE) per sample over time separated per year. Arrows indicate the release dates of the single mass release (blue arrow) or each continuous release (orange arrows).

Figure S2. Mean number of pear psylla (eggs + nymphs) (±SE) per leaf over time separated per year. Arrows indicate the release dates of the single mass release (blue arrow) or each continuous release (orange arrows).

Supplemental Table S1.

Orchard	Location	Date	Product	AI	Rate
Apricot	Yakima Co., WA	May 25th 2021	Genesis Stride 40 WSP	Myclobutanil	350.26g/ha
			Altacor® Insect Control	Chlorantraniliprole-35	280.21g/ha
		June 3rd 2021	Carbaryl 4L	Carbaryl-43	7.01L/ha
		June 15th 2021	Fontelis®	Penthiopyrad-20.4	1.46L/ha
		June 16th 2021	Fontelis®	Penthiopyrad-20.4	1.46L/ha
		July 16th 2021	Isomate® P Pheromone	Pheromone	247 units/ha
		June 24th 2022	Carbaryl 4L	Carbaryl-43	7.01L/ha
		August 9th 2022	Altacor® Insect Control	Chlorantraniliprole-35	280.21g/ha
		August 26th 2022	Delegate® WG Insecticide	Spinetoram-25	420.31g/ha
Cherry	Benton Co., WA	May 17th 2021	GF-120® NF Naturalyte®	Spinosad	1401.06g/ha
		May 21st 2021	GF-120® NF Naturalyte®	Spinosad	1401.06g/ha
			Sulfur Dry Flowable	Sulfur	8.4kg/ha
			Entrust® SC	Spinosad	448.34g/ha
		May 28th 2021	GF-120® NF Naturalyte®	Spinosad	1401.06g/ha
		June 8th 2021	Entrust® SC	Spinosad	448.34g/ha
		June 15th 2021	GF-120® NF Naturalyte®	Spinosad	1401.06g/ha
		May 20th 2022	Sulfur Dry Flowable	Sulfur	8.4kg/ha
			Leprotec®	*Bacillus thuringiensis* ssp. *Kurstaki* strain EVB113-19	3.5L/ha
			Sonata	*Bacillus pumilus* strain QST 2808	9.35L/ha
		May 30th 2022	Sulfur Dry Flowable	Sulfur	8.4kg/ha
			Entrust® SC	Spinosad	448.34g/ha

Orchard	Location	Date	Product	AI	Rate
			Sonata	*Bacillus pumilus* strain QST 2808	9.35L/ha
		June 2nd, 6th, 15th, 22nd, 30th 2022	GF-120® NF Naturalyte®	Spinosad	1401.06g/ha
Pear	Yakima Co., WA	June 7th 2021	Aza-Direct®	Azadirechtin-1.2	3.55L/ha
			IAP 440 Spray Oil	Oil, mineral-98.5	9.35L/ha
		June 21st 2021	Constant BUpH-er	Citric acid and acetic acid	0.58L/ha
			Aza-Direct®	Azadirechtin-1.2	3.55L/ha
			IAP 440 Spray Oil	Oil, mineral-98.5	9.35L/ha
		July 19th 2021	Aza-Direct®	Azadirechtin-1.2	3.55L/ha
			IAP 440 Spray Oil	Oil, mineral-98.5	9.35L/ha
		June 18th 2022	Aza-Direct®	Azadirechtin-1.2	3.5L/ha
			CYD-X® HP Insecticidal Virus	*Cydia pomonella* granulovirus-0.06	0.14L/ha
			Cidetrak® DA MEC	pheromone	29.6ml/ha
			IAP 440 Spray Oil	Oil, mineral-98.5	4.68L/ha
		July 6th 2022	Aza-Direct®	Azadirechtin-1.2	3.5L/ha
			CYD-X® HP Insecticidal Virus	*Cydia pomonella* granulovirus-0.06	0.14L/ha
			Cidetrak® DA MEC	pheromone	29.6ml/ha
			IAP 440 Spray Oil	Oil, mineral-98.5	4.68L/ha
		July 21st 2022	Aza-Direct®	Azadirechtin-1.2	3.5L/ha
			IAP 440 Spray Oil	Oil, mineral-98.5	4.68L/ha
		August 4th 2022	Aza-Direct®	Azadirechtin-1.2	3.5L/ha
			IAP 440 Spray Oil	Oil, mineral-98.5	4.68L/ha
Apple	Yakima Co., WA	June 7th 2021	Serenade® Opti	*Bacillus subtilis* strain QST 713-26.2	1.4kg/ha

Orchard	Location	Date	Product	AI	Rate
			CYD-X® HP Insecticidal Virus	*Cydia pomonella* granulovirus-0.06	0.14L/ha
			Cidetrak® DA MEC	pheromone	29.6ml/ha
		June 22nd 2021	CYD-X® HP Insecticidal Virus	*Cydia pomonella* granulovirus-0.06	0.14L/ha
			IAP 440 Spray Oil	Oil, mineral-98.5	4.68L/ha
		July 1st 2021	Cidetrak® DA MEC	pheromone	29.6ml/ha
			CYD-X® HP Insecticidal Virus	*Cydia pomonella* granulovirus-0.06	0.14L/ha
			IAP 440 Spray Oil	Oil, mineral-98.5	4.68L/ha
		July 17th 2021	CYD-X® HP Insecticidal Virus	*Cydia pomonella* granulovirus-0.06	0.14L/ha
			IAP 440 Spray Oil	Oil, mineral-98.5	4.68L/ha
			Cidetrak® DA MEC	pheromone	29.6ml/ha
		July 26th 2021	IAP 440 Spray Oil	Oil, mineral-98.5	4.68L/ha
			CYD-X® HP Insecticidal Virus	*Cydia pomonella* granulovirus-0.06	0.14L/ha
		August 5th 2021	CYD-X® HP Insecticidal Virus	*Cydia pomonella* granulovirus-0.06	0.14L/ha
			IAP 440 Spray Oil	Oil, mineral-98.5	4.68L/ha
			Cidetrak® DA MEC	pheromone	29.6ml/ha
		July 5th 2022	IAP 440 Spray Oil	Oil, mineral-98.5	4.68L/ha
			CYD-X® HP Insecticidal Virus	*Cydia pomonella* granulovirus-0.06	0.14L/ha
			Aza-Direct®	Azadirechtin-1.2	2.34L/ha
			M-Pede® Insecticide Miticide Fungicide	Potassium salts of fatty acids-49	2.34L/ha
		July 15th 2022	CYD-X® HP Insecticidal Virus	*Cydia pomonella* granulovirus-0.06	0.14L/ha
			IAP 440 Spray Oil	Oil, mineral-98.5	4.68L/ha

Orchard	Location	Date	Product	AI	Rate
			Cidetrak® DA MEC	pheromone	29.6ml/ha
		July 20th 2022	Entrust® SC Naturalyte Insect Control	Spinosad-22.5	0.7L/ha
		September 2nd 2022	CYD-X® HP Insecticidal Virus	*Cydia pomonella* granulovirus-0.06	0.14L/ha
			IAP 440 Spray Oil	Oil, mineral-98.5	4.68L/ha
			Cidetrak® DA MEC	pheromone	29.6ml/ha
		September 10th 2022	CYD-X® HP Insecticidal Virus	*Cydia pomonella* granulovirus-0.06	0.14L/ha
			HI Supreme 440 Spray Oil-NW	Mineral Oil-98.5	4.68L/ha
		September 20th 2022	HI Supreme 440 Spray Oil-NW	Mineral Oil-98.5	4.68L/ha
			CYD-X® HP Insecticidal Virus	*Cydia pomonella* granulovirus-0.06	0.14L/ha
Pear*	Jackson Co., OR	June 17th 2021	Nealta®	Cyflumetofen-18.7	*rates are not known for this orchard
		June 2nd 2022	Gly Star Plus	Glyphosate-41	
			Centrus	Indaziflam-24; Rimsulfuron-16.67	
			Total TNV	Glufosinate-ammonium-24.5	
		June 27th 2022	Intrepid 2F®	Methoxyfenozide-22.6	
		July 7th 2022	Venerate® XC	Heat-killed *Burkholderia* spp. Strain A396	
		July 15th 2022	Ultor	Spirotetramat-14.5	
		August 5th 2022	Altacor®	Chlorantraniliprole-35	
		August 19th 2022	Gly Star Plus	Glyphosate-41	
			Aim EC	Carfentrazone-ethyl-22.3	

Supplemental Table S2.

Apple – WA		Pear – WA		Pear – OR	
**Sampling dates**		**Sampling dates**		**Sampling dates**	
**2021**	**2022**	**2021**	**2022**	**2021**	**2022**
June 21st<br>July 21st<br>August 8th	June 28th<br>July 26th<br>August 25th<br>September 28th	June 17th<br>July 12th<br>August 10th	June 21st<br>July 19th<br>August 16th<br>September 13th	June 6th<br>July 1st<br>July 20th<br>August 9th<br>August 31st	June 8th<br>June 30th<br>July 21st<br>August 11th<br>August 31st

Supplemental Table S3.

	Pears – WA		Pears – OR		Apples – WA	
	Earwigs / trap – pre-counts					
**Treatments**	**2021**	**2022**	**2021**	**2022**	**2021**	**2022**
Control	30.25 (± 2.73)	37.18 (± 3.71)	0	0.02 (± 0.01)	6.4 (±0.76)	13.92 (± 1.27)
Continuous	27.91 (± 3.41)	40.93 (± 6.5)	0	0.04 (± 0.03)	5.31 (± 0.67)	16.31 (± 1.9)
Mass	28.6 (± 4.41)	44.77 (± 5.04)	0	0.24 (± 0.09)	5.4 (± 0.85)	9.88 (±1.52)
χ^2	0.02	0.42	-	2.01	0.04	3.95
df	2	2	-	2	2	2
p	0.98	0.81	-	0.36	0.97	0.13
	**Pear psylla / leaf – pre counts**		**Pear psylla / leaf – pre counts**		**Woolly apple aphid colonies / sample – pre counts**	
	**2021**	**2022**	**2021**	**2022**	**2021**	**2022**
Control	0.84 (± 0.14)	0.13 (± 0.03)	0.01 (± 0.008)	0.03 (± 0.01)	4.65 (± 0.54)	0.64 (± 0.12)
Continuous	0.64 (± 0.21)	0.09 (± 0.03)	0	0	4.51 (± 0.57)	0.95 (± 0.25)
Mass	0.97 (± 0.26)	0.11 (± 0.02)	0.007 (± 0.007)	0.015 (± 0.015)	3.17 (± 0.5)	0.4 (± 0.12)
χ^2	1.66	0.65	0.7	1.4	2.04	0.57
df	2	2	2	2	2	2
p	0.43	0.72	0.7	0.5	0.36	0.75

Supplemental Table S4.

	Pears – WA				Pears – OR				Pears – WA only				Apples – WA			
	Twospotted spider mites / leaf		Pear rust mites / leaf		Twospotted spider mites / leaf		Pear rust mites / leaf		European red mites / leaf		Brown mites / leaf		Green apple aphid colonies / sample		Rosy apple aphid colonies / sample	
**Treatments**	**2021**	**2022**	**2021**	**2022**	**2021**	**2022**	**2021**	**2022**	**2022**	**2022**	**2021**	**2022**	**2021**	**2022**	**2021**	**2022**
Control	0.06 (± 0.02)	0.008 (± 0.004)	0.12 (± 0.05)	0	0.09 (± 0.006)	0.01 (± 0.004)	0	0	0.81 (± 0.16)	0.48 (± 0.12)	0.3 (±0.08)	0	0.29 (±0.1)	0.001 (± 0.001)	0.03 (±0.02)	0
Continuous	0.05 (± 0.01)	0.006 (± 0.004)	0.16 (± 0.12)	0	0.01 (± 0.009)	0.03 (± 0.01)	0	0	0.76 (± 0.2)	0.2 (± 0.05)	0.21 (±0.07)	0	0.18 (±0.07)	0	0.11 (±0.07)	0.003 (± 0.003)
Mass	0.03 (± 0.01)	0.001 (± 0.001)	0.1 (± 0.05)	0	0.01 (± 0.007)	0.03 (± 0.01)	0	0	0.52 (± 0.14)	0.26 (± 0.05)	0.27 (± 0.07)	0	0.01 (± 0.01)	0.02 (±0.01)	0	0.003 (± 0.0003)
χ^2	1.01	1.15	0.03	-	0.84	1.76	-	-	0.43	0.46	0.2	-	2.91	2.21	0.18	-
df	2	2	2	-	2	2	-	-	2	2	2	-	2	2	2	-
p	0.6	0.56	0.98	-	0.65	0.41	-	-	0.8	0.79	0.9	-	0.23	0.33	0.91	-

Supplemental Figure S1.

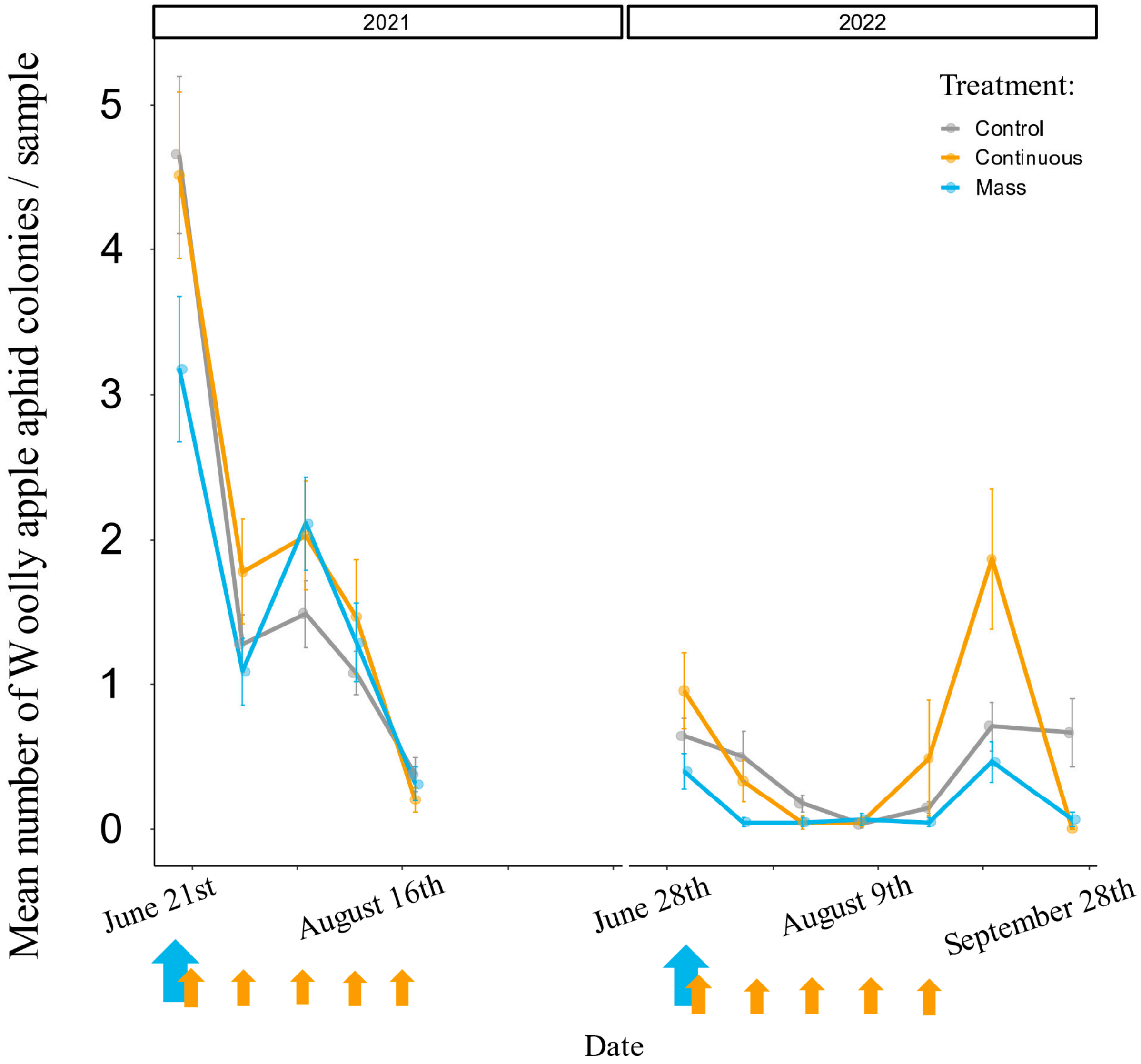

Supplemental Figure S2.

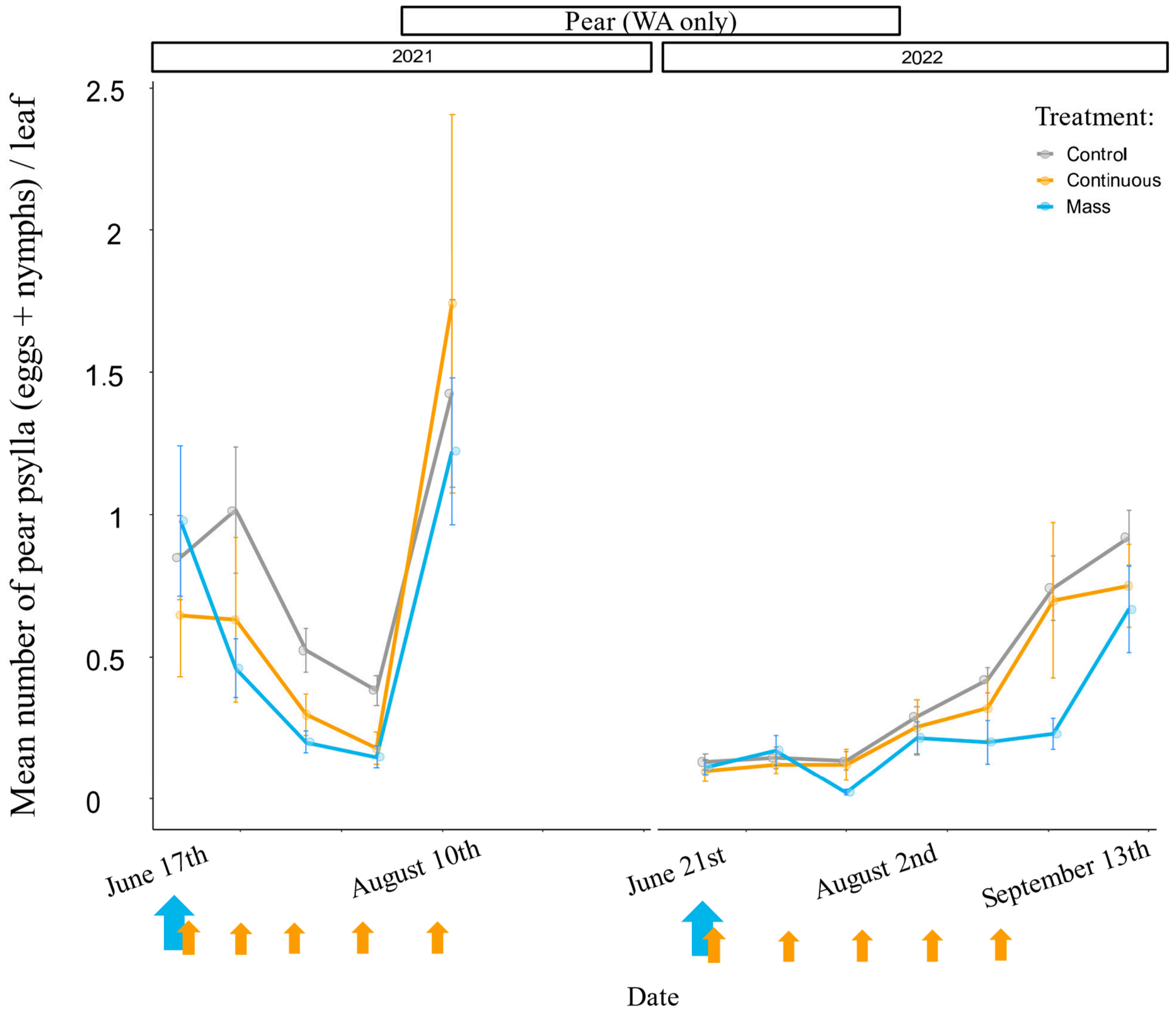

Supplement: Supplementary file 1 [file insects-14-00906-s001.zip › Supplemental materials.pdf]
